# *ESKIMO1 *is a key gene involved in water economy as well as cold acclimation and salt tolerance

**DOI:** 10.1186/1471-2229-8-125

**Published:** 2008-12-07

**Authors:** Oumaya Bouchabke-Coussa, Marie-Luce Quashie, Jose Seoane-Redondo, Marie-Noelle Fortabat, Carine Gery, Agnes Yu, Daphné Linderme, Jacques Trouverie, Fabienne Granier, Evelyne Téoulé, Mylène Durand-Tardif

**Affiliations:** 1 Cell Biology Laboratory, IJPB, INRA-CIRAD, UR0501, Route de St Cyr, F-78026 Versailles, France; 2 Physiology and Biotechnologies Laboratory, Faculty of Sciences, University of Lomé BP 1515 Lomé, Togo; 3 Danmarks Tekniske Universitet, Institut for Vand og Miljøteknologi, Bygningstorvet, B115, DK-2800 KGS. Lyngby, Danmark; 4 URGV, Plant Genomics Research Unit, INRA/CNRS, UMR11, 2 rue Gaston Crémieux CP5708, F-91057 Evry, France; 5 CIRAD, Pôle de Protection des Plantes, Ligne Paradis, F-97410 St Pierre, France; 6 Variability and Abiotic Stress Tolerance, Genetics and Plant Breeding Laboratory, IJPB, INRA, UR0254, Route de St Cyr, F-78026 Versailles, France

## Abstract

**Background:**

Drought is a major social and economic problem resulting in huge yield reduction in the field. Today's challenge is to develop plants with reduced water requirements and stable yields in fluctuating environmental conditions. *Arabidopsis thaliana *is an excellent model for identifying potential targets for plant breeding. Drought tolerance in the field was successfully conferred to crops by transferring genes from this model species. While involved in a plant genomics programme, which aims to identify new genes responsible for plant response to abiotic stress, we identified *ESKIMO1 *as a key gene involved in plant water economy as well as cold acclimation and salt tolerance.

**Results:**

All *esk1 *mutants were more tolerant to freezing, after acclimation, than their wild type counterpart. *esk1 *mutants also showed increased tolerance to mild water deficit for all traits measured. The mutant's improved tolerance to reduced water supply may be explained by its lower transpiration rate and better water use efficiency (WUE), which was assessed by carbon isotope discrimination and gas exchange measurements. *esk1 *alleles were also shown to be more tolerant to salt stress.

Transcriptomic analysis of one mutant line and its wild-type background was carried out. Under control watering conditions a number of genes were differentially expressed between the mutant and the wild type whereas under mild drought stress this list of genes was reduced. Among the genes that were differentially expressed between the wild type and mutant, two functional categories related to the response to stress or biotic and abiotic stimulus were over-represented. Under salt stress conditions, all gene functional categories were represented equally in both the mutant and wild type. Based on this transcriptome analysis we hypothesise that in control conditions the *esk1 *mutant behaves as if it was exposed to drought stress.

**Conclusion:**

Overall our findings suggest that the *ESKIMO1 *gene plays a major role in plant response to water shortage and in whole plant water economy. Further experiments are being undertaken to elucidate the function of the ESKIMO1 protein and the way it modulates plant water uptake.

## Background

Understanding plant response to abiotic stress is of interest to both basic and applied research. Recently, our knowledge of the mechanisms developed by plants to sense and transfer stress signals, and then orchestrate gene expression in order to protect and/or repair tissues and cells, made rapid progress [1]. Nevertheless, many questions regarding these mechanisms, which are of great importance in biology, remain to be answered. At the same time, maintaining agricultural supply in a fluctuating environment is a major challenge for the XXI^st ^century. Crop yield losses induced by environmental stress are estimated to reach 60–70% [2,3]. A major challenge over the coming decades is to develop plant varieties with reduced requirements for water and other inputs and which also maintain stable yields in diverse environmental conditions.

The overall response by plants to environmental constraints has been well characterised and extensively reviewed [1,4-8]. Stress from the environment leads to both specific and common effects and responses. Drought is particularly complex because it leads to simultaneous physiological responses at the whole plant, cellular and molecular levels. For example, drought induces mechanical stress on roots due to soil hardness [9], osmotic stress because of cell dehydration and removal of water to the extra-cellular space [10], and oxidative stress by the accumulation of reactive oxygen species (ROS) [11]. During cold and salt stress the physiological response is similar to that caused by drought [12,13], meaning that the effects of different environmental stresses are tightly interconnected.

Stress sensing is still an unknown process: the nature of the first physical or chemical signal remains hypothetical [14]. Signal transduction is better understood, but remains complex because of the crosstalk between different signalling pathways [15]. It involves diverse molecular mechanisms such as protein phosphorylation [16], modifications to membrane phospholipids which affect membrane fluidity and release signal molecules such as inositol triphosphate (IP_3_) and changes Ca^2+^concentration in the cytosol [17]... Drought and salt stress trigger ABA production, which in turn induces the expression of a number of responsive genes. Many but not all stress response genes respond to ABA [18,19]. ROS can also be important signalling molecules [11,20,21], and stimulate Ca^2+^, ABA and MAPK cascades.

Genes induced by stress can be roughly classified into two groups: genes coding for regulatory proteins, mainly transcription factors, and genes encoding proteins involved directly in response mechanisms; genes from both classes are of interest. Variations in the expression of regulators could lead to a protective status before the emergence of stress and have multiple effects. Genes involved in protection or repair mechanisms could be new targets for the improvement of plant plasticity and adaptive responses to stress [22]. The unraveling of general stress responses in the model species *Arabidopsis thaliana *helped to identify potential targets for plant breeding. Arabidopsis genes involved in tolerance to abiotic stress were transferred, by genetic engineering, to many crops and tolerance was successfully conferred in the field, despite the complexity of plant responses to environmental stress [23-28]. Thus, finding new key genes responsible for abiotic stress tolerance phenotypes is of great importance not only for a better understanding of stress responses, but also for promising future crop improvement.

Our team is involved in a plant genomics programme where a series of candidate genes was analysed for their role in environmental stress responses, using *Arabidopsis thaliana *insertional mutants [29]. A list of candidate genes and corresponding mutants was compiled by an *in silico *search for Arabidopsis genes with homology to maize and/or wheat genes which showed modified expression in response to water deficit, salt or cold stress . A mutant line in the *ESKIMO1 *gene was retained both in the cold and drought screens because it responds to stress differently to wild type. Initially Xin and Browse [30] identified the *eskimo1 *mutation as conferring freezing tolerance without cold acclimation. They observed that a significantly high proline content accumulates in *esk1 *mutants as a mechanism to balance the osmotic stress. Ghars *et al*. [31] observed a similar proline content in wild type and *esk1 *mutant, but proline accumulation was higher in *esk1 *in response to salt stress. Xin *et al*. [32] identified the *eskimo1 *mutation by positional cloning. The gene product belongs to an uncharacterised plant-specific protein family containing 48 members. Bioinformatics analysis of genes whose expression was modified by the *eskimo1 *mutation showed that a large number were previously reported to be induced by salt, osmotic stress and the stress hormone ABA, however Xin *et al*. did not consider that the mutant is drought or salt tolerant.

In this article, we describe the response by *ESK1 *allelic mutants to different abiotic stresses in two genetic backgrounds (WS and Col-0). We found that the mutant lines have a clear advantage in response to drought and salt stress, but at the cost of biomass production. Nevertheless, this cost could be compensated by the maintenance of growth over a large range of environmental conditions. Based on physiological tests and transcriptomic analysis we could formulate a hypothesis regarding ESKIMO1 function. Our results are discussed in relation to those reported by Xin and coworkers [32].

## Results

### Characterisation of the *esk1-6 *mutant line

#### • Without abiotic constraint

**Figure 1:**
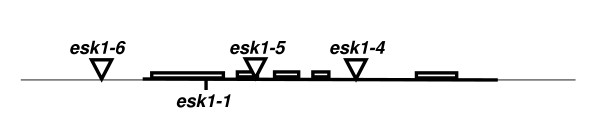
**Schematic representation of the *ESKIMO1 *gene**. The thin line represents the genomic DNA and the bold line is the *ESKIMO1 *mRNA, bold boxes represent exons. The triangles indicate the insertion sites for the mutant lines. The position of the *esk1-1 *mutation described by Xin and collaborators is also indicated. The *ESKIMO1 *gene is 2919 nucleotides long.

*Esk1-6 *is a line with an insertion in the promoter of the *ESK1 *gene (Figure 1) in the WS genetic background. In the absence of drought constraints, *esk1-6 *has the same phenology as its wild type genetic background. It undergoes normal development, and produces the same number of leaves which are normal in shape and colour. However, it differs from WS in some general characteristics such as plant size and tissue water content (Table 1). Indeed, 7 days after 6^th ^leaf emergence, the Total Leaf Area (TLA) of the *esk1-6 *mutant was 1.5 times smaller than that of wild type. The rosette fresh weight (FW) and dry weight were also smaller. Under the same conditions, the relative water content (RWC) of *esk1-6 *rosette was 10% lower than in the wild type (Table 1).

**Table 1:** General characterization of the *esk1 *mutant lines

	**Mean TLA** **(cm^2^)**	**SD**	**Mean FW** **(mg)**	**SD**	**Mean DW** **(mg)**	**SD**	**Mean RWC** **(g_W_g_DW_^-1^)**	**SD**
**WS (WT)**	**8.70**	**0.78**	**223**	**36**	**19.51**	**3.19**	**10.45**	**0.19**

***esk1-6***	6.02 (69%)	1.13	148 (66%)	20	13.83 (71%)	1.96	9.70 (93%)	0.33

**Col-0 (WT)**	**6.72**	**0.95**	**170**	**24**	**15.76**	**1.42**	**10.72**	**0.31**

***esk1-4***	2.87 (43%)	0.46	60 (35%)	6	7.27 (46%)	0.65	8.18 (76%)	0.12

***esk1-5***	3.53 (53%)	0.64	69 (41%)	9	8.77 (56%)	1.11	7.93 (74%)	0.34

Measurements of Total Leaf Area (TLA, cm^2^), Fresh Weight (FW, mg), Dry Weight (DW, mg), and Relative Water Content (RWC, g_W_g_DW_^-1^), for the *esk1-6 *mutant line and its wild type genetic background, WS, and the *esk1-4 *and *esk1-5 *mutant lines and their wild type genetic background, Col-0. Each measurement was made on plants under monitored control conditions i.e. in the absence of drought constraint. The percentage of each mutant value compared to the value for wild type is given in brackets. SD: Standard Deviation. All the mutant values were significantly different from the wild type values (P < 0.05).

#### • Response to monitored mild water-deficit

**Figure 2:**
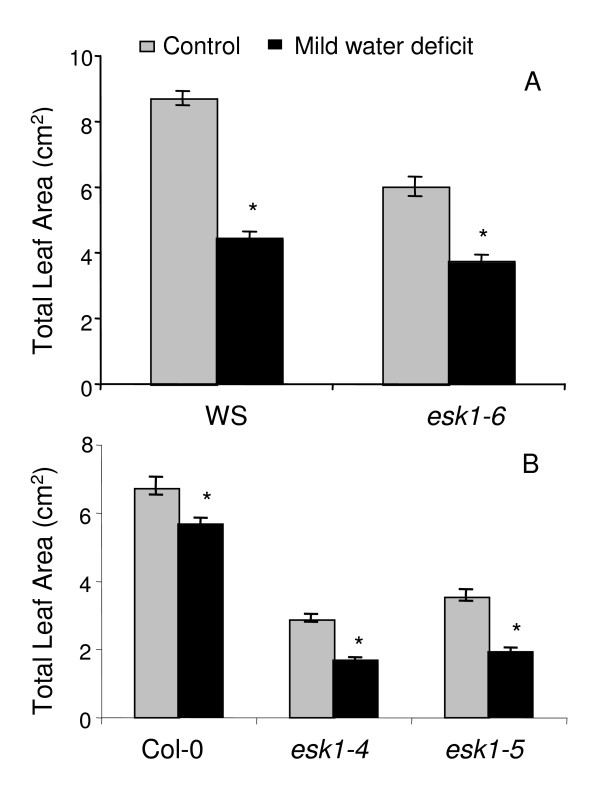
**Total Leaf Area of *esk1 *mutant lines and wild type**. Total Leaf Area (TLA, cm^2^), calculated for the *esk1-6 *homozygous mutant line and WS wild-type (A), as well as *esk1-4 *and *esk1-5 *homozygous mutant lines and Col-0 wild-type (B), in control (grey bars) and mild water deficit (black bars) conditions, determined using the averaged approach as described in Table 2. Stress was induced in plants at growth stage 1.07 for (A) and at 1.06 for (B). Measurements were made on 10 plants of each genotype and in each condition. Error bars are standard errors. * indicates a significant statistical difference (P < 0.05) between control and water deficit conditions.

In a first experiment, TLA was calculated for the homozygous mutant and wild type after 7 days of averaged substrate water content (SWC): 60% for control and 30% for mild water deficit (Table 2). The TLA of the *esk1-6 *mutant was reduced by 38% compared to the TLA in control conditions whereas the wild type TLA was reduced by almost 50% (Figure 2A).

**Table 2:** Monitored stress: experimental system on propagation plugs

At time 0, propagation plug saturation was 100% (SMWC = Substrate maximum water content)			
**Propagation plug saturation**	**60%** **Control**	**30%** **Mild water deficit**	**20%** **Severe water deficit**

**Averaged determination**	All the plants were watered daily to reach 60% SMWC with a volume based on the average weight of a subset of propagation plugs	All the plants were watered daily to reach 30% SMWC with a volume based on the average weight of a subset of propagation plugs	*Not done (threshold effect)*

**Individual determination**	Each plant was watered daily to reach 60% SMWC based on the actual weight of the propagation plug	Each plant was watered daily to reach 30% SMWC based on the actual weight of the propagation plug	Each plant was watered daily to reach 20% SMWC based on the actual weight of the propagation plug

**Salt stress: individual determination**	Each plant was watered daily to reach 60% SMWC based on the actual weight of the propagation plug. Once at 60%, salt stress was applied with 0.5× a nutritive solution supplemented with 150 mM NaCl.	*Not done (combined water deficit and salt stress not applied)*	*Not done (combined water deficit and salt stress not applied)*

In a second experiment, we measured the TLA of the segregating T3 *esk1-6 *population (progeny testing) under averaged mild water deficit and control conditions. Plant samples were harvested after 9 days of treatment and genotyped. The groups were phenotyped by determining their TLA and the results are presented in Table 3. Segregation analysis of the insertion in the *ESK1 *gene resulted in 56% heterozygous, 24% wild type and 20% homozygous plants. Thus, the tests converged to indicate a recessive knock-out mutation, even considering that the insertion lies in the gene promoter.

**Table 3:** Total Leaf Area analysis of the segregating *esk1-6 *T3 population

	**Control**	**Water deficit**
Heterozygous/wt	NS (4.42/3.82)	NS (2.81/2.97)

Heterozygous/homozygous	S*** (4.42/2.99)	S*** (2.81/1.52)

Wt/homozygous	S* (3.82/2.99)	S*** (2.97/1.52)

Monitored control and mild water deficit stress (averaged determination) was applied to a segregating T3 population of *esk1-6 *mutant plants (203 individuals). A one factor ANOVA was performed to test the significance of the difference in TLA between heterozygous vs. wild type, heterozygous vs. homozygous and wild type vs. homozygous genotypes. TLA values (cm^2^) for each genotype for each treatment are given in brackets. NS: non significant; S*: significant with 0.05 > p-value > 0.01; S***: significant with p-value < 0.001.

**Figure 3:**
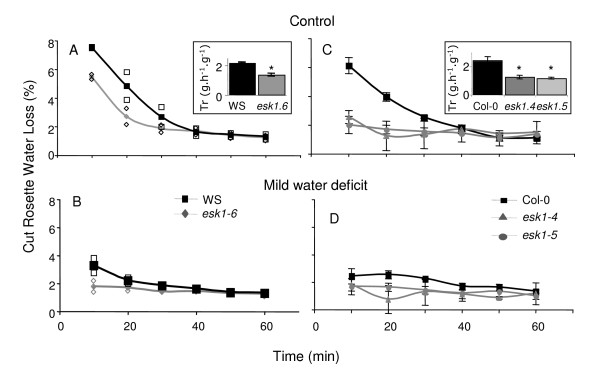
**Cut Rosette Water Loss of *esk1 *homozygous mutant lines and wild type**. Cut Rosette Water Loss (CRWL, %) was determined for the *esk1-6 *homozygous mutant line (grey) and WS wild-type (black), in control (A) and mild water deficit conditions (B) (averaged determination method, table 2), and for *esk1-4 *(grey triangles) and *esk1-5 *(grey circles) homozygous mutant lines and Col-0 wild-type (black), in control (C) and mild water deficit conditions (D) (averaged determination method, table 2). For (A) and (B), measurements were made on two replicates of 5 rosettes each that are plotted (empty squares for Col-0 and empty diamonds for *esk1-6*); the curves show the means. For (C) and (D) measurements were made on five replicates of two rosettes each; the curves show the means and error bars represent standard errors. Weights were measured every 10 minutes. Inset: real transpiration measured every hour for a 6 hours period, in averaged control conditions. Each bar represents the mean data of 5 plants. Error bars are standard errors. * indicates a significant statistical difference P < 0.05 between wild type and mutants.

Cut rosette water loss (CRWL i.e. water loss/fresh weight) measurements were carried out on plants that were submitted to mild water deficit and control conditions (averaged determination method). Results are shown in Figure 3A for the control and Figure 3B for the mild water deficit conditions. In both treatments, the *esk1-6 *homozygous mutant line had a lower CRWL value than the wild type, in the first 40 minutes. The reduced levels of mutant water loss were confirmed in transpiration experiments *in planta*. Indeed, *esk1-6 *showed lower transpiration rates than the wild type in standard and mild drought stress conditions (Figure 3A, inset p < 0.05).

#### • Response to cold

We performed cold tests on plantlets in soil to be as close as possible to field conditions. The cold tolerance test was first performed on the segregating T3 *esk1-6 *population. Contrasted levels of resistance were scored suggesting that the mutant was behaving differently from wild type. Then, homozygous *esk1-6 *lines were subjected to the freezing test and the percentage of viable plants scored. The mutant was more tolerant than WS when exposed to freezing after acclimation (Table 4). However, *esk1-6 *did not show a significant level of tolerance to freezing without previous acclimation.

**Table 4:** Viability of the wild-type genetic background and respective mutants after freezing.

**Mutant line**	**% viability mutant**	**(wild type) % viability wt**	**p-value**
***esk1-4***	8.82	(Col-0) 1.82	S*

***esk1-5***	42.11	(Col-0) 10.43	S***

***esk1-1***	40.69	(Col-0) 6.84	S***

***esk1-6***	16.76	(WS) 6.52	S**

Plants were acclimated for 7 days at 5°C and then exposed to -8°C for 48H. % of viability was scored as described in the methods. S*: significant with 0.05 > p-value > 0.01; S**: significant with 0.01 > p-value > 0.001; S***: significant with p-value < 0.001.

#### • Response to osmotic stress

**Figure 4:**
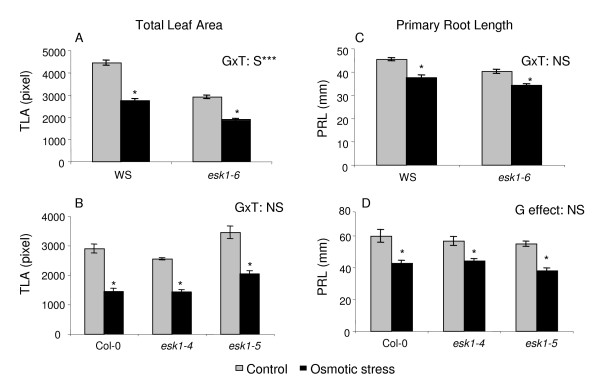
**Response to osmotic stress by *esk1 *mutant lines and wild type**. Graphs show the Total Leaf Area (TLA in pixels) and Primary Root Length (PRL in mm) of the *esk1-6 *homozygous mutant line and WS wild type (A and C) and of *esk1-4 *and *esk1-5 *homozygous mutant lines and Col-0 wild type (B and D) on control media or media supplemented with 60 mM (A and C) or 75 mM (B and D) mannitol. * indicates that a significant treatment effect was observed on all the traits measured. GxT: S*** indicates that there was a significant genotype × treatment interaction with a p-value < 0.001. GxT: NS indicates that there was no significant genotype × treatment interaction. G effect: NS indicates that there was no significant genotype effect in control and in stress conditions. Error bars are standard errors.

Next, osmotic stress was applied *in vitro *on WS and *esk1-6 *lines with 60 mM mannitol. The primary root length (PRL) and TLA were assessed on plantlets (Figure 4A and 4C).

We observed that the treatment and genotype had a significant effect on the TLA and PRL (p < 0,001) but a significant genotype × treatment interaction was only seen for the TLA (p < 0,001).

### Analysis of *esk1 *alleles in the Col-0 genetic background

#### • Without abiotic constraint

**Figure 5:**
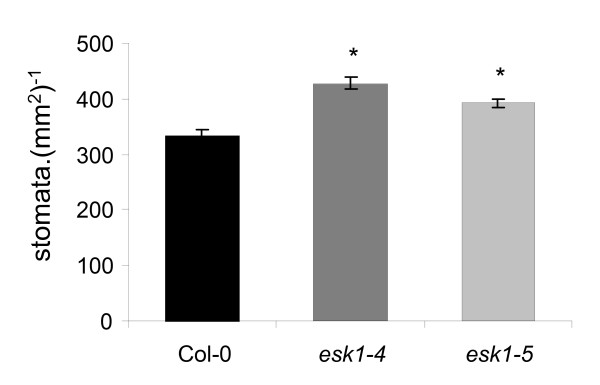
**Stomatal density for *esk1 *mutant lines and wild type**. The graph shows the number of stomata per mm^2^. Measurements were made as described in the Material and Methods. Error bars are standard errors. * indicates a significant statistical difference (P < 0.05) between mutant lines and wild type Col-0.

In order to strengthen the results obtained, we also analysed independent insertional mutant lines in the ESK1 gene in a second genetic background. The *esk1-4 *(SALK_078275) and *esk1-5 *(SALK_089531) homozygous mutant lines were analysed as well as Col-0, their wild type genetic background. When growth was observed in our standard growth conditions, in propagation plugs, there were no statistically significant differences between the time of bolting of Col-0, *esk1-4 *and *esk1-5 *plants and the overall phenology was identical for the three lines. When the other parameters (TLA, FW, DW, RWC; Table 1 and CRWL; Figure 3) were examined as previously, there were no significant differences between the *esk1-4 *and *esk1-5 *lines. At the end of the vegetative phase, however, the TLA of the mutant lines was 2 times smaller than that of the wild type. Of particular note, the Relative Water Content of the mutant lines was significantly lower than in Col-0. The number of stomata per leaf area was significantly higher for both mutants (282 for *esk1-4 *and 259 for *esk1-5*) than the wild type (220, Figure 5). The number of stomata per leaf area was not significantly different between *esk1-4 *and *esk1-5*. Finally, we observed that *esk1-4 *produced half as many seeds as Col-0, and *esk1-5 *produced one third as many as Col-0 (data not shown).

#### • Response to cold

Homozygous *esk1-4 *and *esk1-5 *lines were subjected to the freezing test described in the Methods section and the viability was scored. Both mutants exhibited higher tolerance than Col-0, when exposed to freezing after acclimation (Table 4). However without previous acclimation, all the plants of both the mutant lines and wild type died.

#### • Response to mild water deficit

TLA was calculated for the homozygous mutant lines and wild type after 7 days of averaged mild water deficit and control conditions (Table 2). The TLAs of the *esk1-4 *and *esk1-5 *mutant lines were reduced by 42% and 46% respectively compared to the TLA in standard conditions whereas the wild type TLA was reduced by 16% (Figure 2B).

#### • Response to salt

We observed germination and plantlet growth *in vitro *on control medium and medium supplemented with 100 mM, 150 mM and 200 mM NaCl. The NaCl concentration had no effect on germination but did have a significant effect on root and leaf growth (treatment effect), but the mutant lines and Col-0 responded in the same way and there was no genotype × treatment interaction (data not shown).

**Figure 6:**
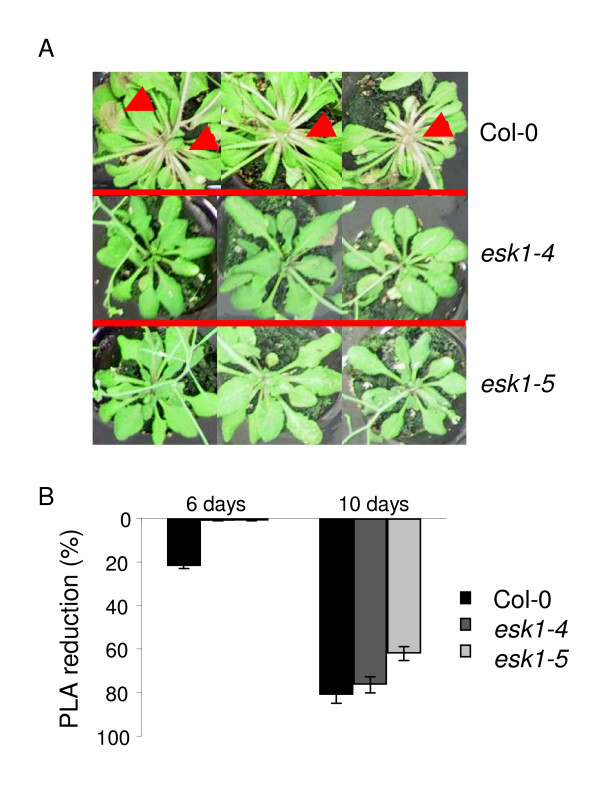
**Response to salt stress by *esk1 *mutant lines and wild type**. The photo shows three Col-0 (1^st ^line), *esk1-4 *(2d line) and *esk1-5 *(3^rd ^line) plants after soaking for 24 hours in concentrated nutritive solution. Red arrows indicate areas where photosynthetic tissues are being lost. The graph shows the % of PLA reduction for the mutant lines *esk1-4 *(grey bars) and *esk1-5 *(light grey bars) and Col-0 (black bars) after 6 (three bars on the left) and 10 days (three bars on the right) of salt stress as described in the Material and Methods. Error bars show the 95% confidence interval.

We also tested the effect of a nutritive solution supplemented with 200 mM NaCl, on plants grown in pots. Important phenotypic differences were observed for vegetative organs after 6 days of treatment. Compared to plants under standard watering regime, the Photosynthetic Leaf Area (PLA or Total Leaf Area minus chlorotic area) was reduced by more than 20% for Col-0 but no significant reduction was observed for the *esk1-4 *and *esk1-5 *lines (Figure 6). After 10 days of treatment, the Col-0 PLA was reduced by more than 80%, the leaves were dry even if the plants were still alive. For the *esk1-4 *mutant line, after 10 days of treatment the PLA reduction reached 76% compared to plants grown in standard conditions and for *esk1-5 *mutant lines there was a reduction of 62%. Neither of the mutant lines had wilted and they had green photosynthetic leaves.

#### • Response to osmotic stress

Osmotic stress was applied *in vitro*. Col-0, *esk1-4 *and *esk1-5 *were grown on standard medium or medium supplemented with 75 mM mannitol. TLA and PRL were assessed on plantlets (Figure 4B and 4D). We observed a treatment effect in all the analysis. There was no significant genotype effect on PRL, in control or osmotic stress conditions. There was a genotype effect on TLA for *esk1-5 *only, in control and osmotic stress conditions.

#### • Water starvation

**Figure 7:**
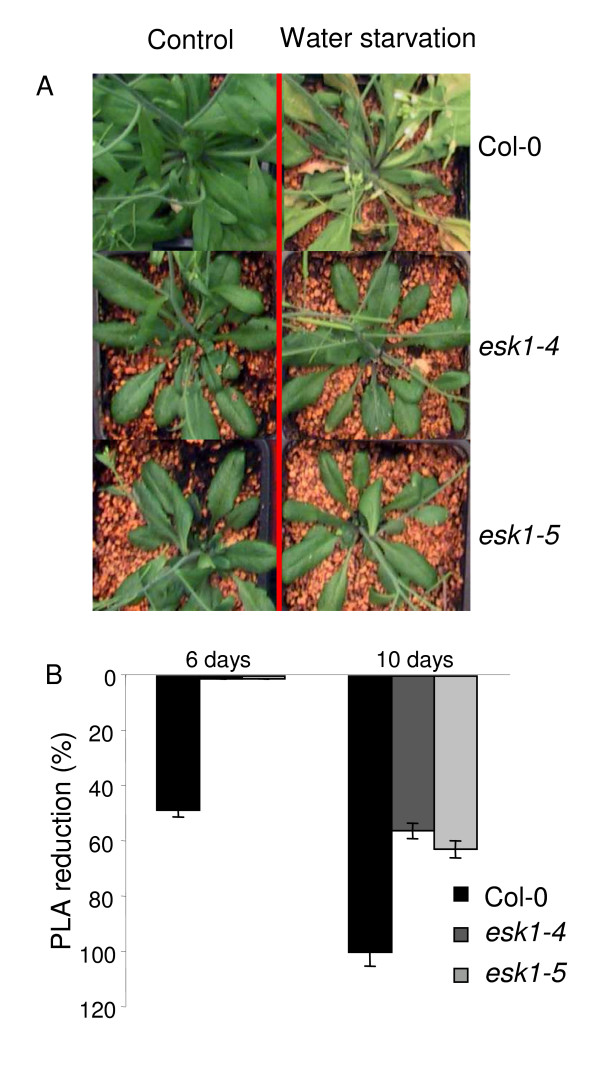
**Response to water starvation by *esk1 *mutant lines and wild type – A**. The photo shows Col-0 (1^st ^line), *esk1-4 *(2d line) and *esk1-5 *(3^rd ^line) plants after 6 days of standard watering (left side) or water starvation (right side). The graph shows the % of PLA reduction for the mutant lines *esk1-4 *(grey bars) and *esk1-5 *(light grey bars) and wild type Col-0 (black bars) after 6 (three bars on the left) and 10 days (three bars on the right) of water starvation as described in the Material and Methods. Error bars show the 95% confidence interval.

To determine the response of the *esk1-4 *and *esk1-5 *mutant lines to water starvation, plants were grown in pots until the reproductive stage and then subjected to a 10-day water-starvation period (Figure 7A and 7B). Six days after water starvation, the wild type plants showed a withering phenotype. At this stage, the Col-0 PLA had already decreased by half compared to watered plants, on the other hand, the two mutant lines were not wilting. After 10 days of water-starvation, the mutants PLA had decreased by between 55% and 63%, while their leaves remained green. At day 10, the leaves of wild type plants were almost completely dry.

**Figure 8:**
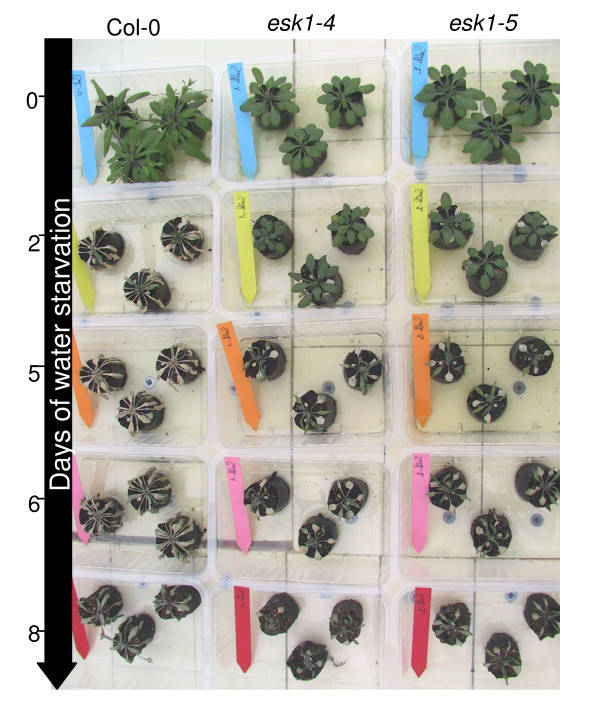
**Response to water starvation by *esk1 *mutant lines and wild type – B**. The photo shows Col-0 (1^st ^column), *esk1-4 *(2d column) and *esk1-5 *(3^rd ^column) plants in Fertiss^® ^propagation plugs after 0 (blue tags), 2 (yellow tags), 5 (orange tags), 6 (pink tags) or 8 (red tags) days of water starvation and re-watering with 0.5× nutritive solution.

A second experiment was carried out on Col-0, *esk1-4 *and *esk1-5 *plants grown in propagation plugs in the greenhouse. After 2, 5, 6 or 8 days of water-starvation, the plants were re-watered every day with 0.5× nutritive solution. Thus, we could observe the response of the different genotypes to drought stress followed by re-watering. After 2-days of water starvation, the Col-0 plants became dry and only 50% survived after the re-watering. On the contrary, *esk1-4 *and *esk1-5 *plants did not seem to be affected by this stress (Figure 8). After 5-days of water starvation, the Col-0 plants were all dead, whereas 100% of the *esk1-4 *and *esk1-5 *plants survived. Only some white spots appeared on their leaves, showing local tissue degradation. After 6-days, some *esk1-4 *and *esk1-5 *plants died. Finally, after 8-days of water starvation, none of the plants survived (for the wild type or mutant genotypes) under these experimental conditions.

#### • Water consumption and Water Use Efficiency (WUE)

**Figure 9:**
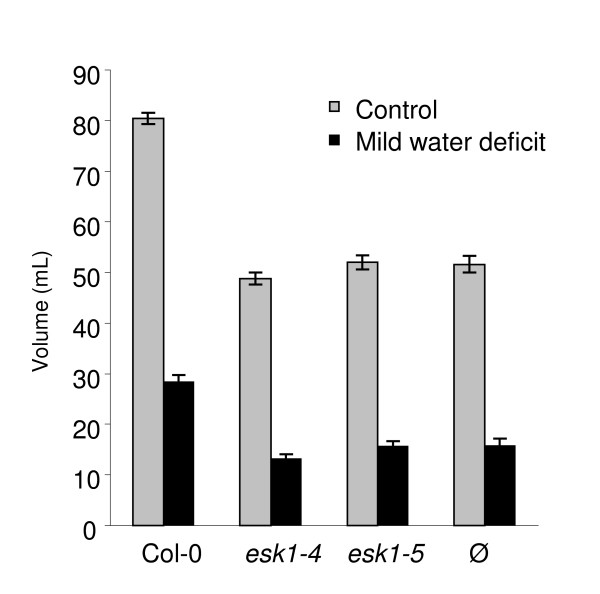
**Water usage by *esk1-4*, *esk1-5 *and wild type**. Plants were grown as described in Table 2: saturation in the propagation plugs was determined individually and maintained at 60% (control) and 30% (mild water deficit). The graph shows the means of the total amount of 0.5× nutritive solution with which plants were watered to reach and maintain control (grey bars) or mild water deficit conditions (black bars) from day 0 to day 9. Each data point was derived from ten replicates, for propagation plugs with (Col-0, *esk1-4*, *esk1-5*) or without plants (Ø). Error bars are standard errors.

We measured the daily quantity of nutritive solution necessary to maintain the three genotypes, Col-0, *esk1-4 *and *esk1-5*, to targeted levels of 60 and 30% saturation in the propagation plugs. We applied the individual determination method for control and mild water deficit treatments according to our experimental chart (Table 2). Propagation plugs where rosettes were removed, were also included in the experiment to assess substrate evaporation under the culture conditions. The experiment started at bolting. Figure 9 shows the total amount of nutritive solution added during the course of the experiment from day 0 to day 9. The difference in water consumption between Col-0 and the *esk1-4 *and *esk1-5 *lines was significant, with the mutants consuming less water than wild type. There was no detectable difference in water consumption/evaporation between *esk1-4*, *esk1-5 *and empty propagation plugs, although plants were still developing floral stems.

**Figure 10:**
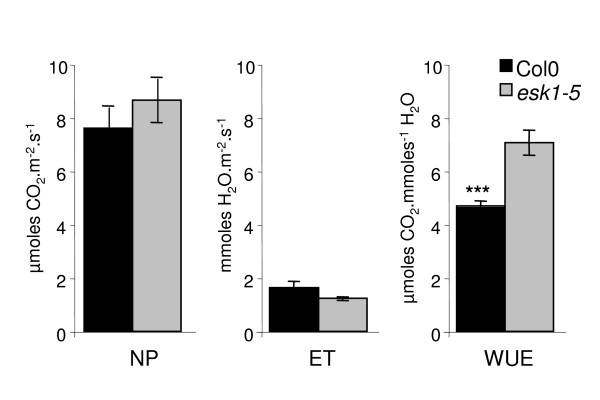
**Water Use Efficiency in *esk1-5 *and wild type**. NP is the net photosynthesis (μmoles CO_2_.m^-2^.s^-1^) and ET is Evapo-Transpiration (mmoles H_2_O.m^-2^.s^-1^) calculated from gas exchanges measurements using a portable gas exchange system (Li-6400; LI-COR^®^). WUE was estimated as the ratio of net photosynthesis to evapo-transpiration. Each bar represents the mean value of three independent measurements for each genotype. Error bars are standard errors. *** indicates a significant statistical difference P < 0.001 between wild type and the *esk1-5 *mutant.

Next, we estimated the water use efficiency (WUE: aerial biomass synthesised/water consumed) by first measuring CO_2 _consumption and H_2_O release with a portable gas exchange system. Wild type and *esk1-5 *plants were grown in monitored control conditions. Results are shown in Figure 10 which clearly shows that the *esk1-5 *WUE was significantly higher than the Col-0 WUE (33.6%).

**Figure 11:**
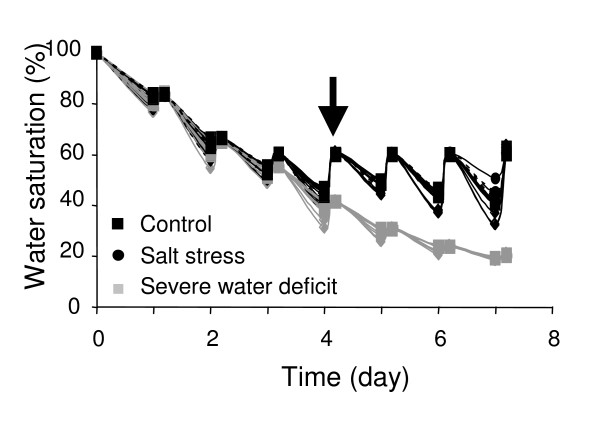
**Experimental system for transcriptome and Water Use Efficiency**. The graph shows propagation plugs water saturation throughout the experiment (see Table 2). Black curves show plug water content in control and salt stress conditions; grey curves show plug water content in severe drought stress conditions. From day 4, 0.5× nutritive solution supplemented with NaCl 150 mM was applied to plants under the salt stress conditions.

WUE was also evaluated by carbon isotope discrimination. Control, mild water-deficit and salt stressed plants were cultivated using the individual determination method for soil water content, as described in the Material and Methods (Table 2, Figure 11). Because at day 4 the water content of the propagation plugs was different between the control and the drought stressed plants, salt stress was induced by saturating the propagation plugs at 60% with a 0.5× nutritive solution supplemented with 150 mM NaCl. Rosettes were harvested at day 7. Results are shown in Table 5. There was an obvious difference in the carbon isotope composition (≅10%), in the three conditions, between wild type and mutant plants. Salt stress did not significantly change the carbon isotope composition compared to control conditions but drought stress did. The WUE improved to a similar extent under water deficit for the three genotypes compared to control conditions: the carbon isotope discrimination value was approximately 10% higher for the two allelic mutants than the wild type under control or drought stress condition.

**Table 5:** Carbon isotopes discrimination for *esk1-4*, *esk1-5 *and wild type

	**Control**		**Water deficit**		**Salt**	
**Genotype**	Mean (‰)	SE	Mean (‰)	SE	Mean (‰)	SE

**Col-0**	32.29	0.36	30.97	0.57	32.09	0.04

***esk1-4***	29.21	0.28	28.66	0.23	29.03	0.32

***esk1-5***	29.35	0.11	28.58	0.06	28.99	0.11

Wild type (Col-0), *esk1-4 *and *esk1-5 *mutant lines were grown under individually determined control, severe water deficit or salt stress conditions. δ^13^C values correspond to the mean of 2 replicates, each representing a pool of three plants. SE: Standard Error.

#### • Transcriptome analysis of the *esk1-5 *mutant line and Col-0

The Col-0 and *esk1-5 *transcriptome was analysed by individually determined control, severe drought and salt stress conditions (Table 2). The experimental conditions were the same as those used for WUE assessment (Figure 11). Two biological replicates were used. Each replicate was a pool of three plants. For this study, data were normalized and the p-value was adjusted using the Bonferroni method, with a 0.05 threshold. Among the Gene Sequence Tags (GST) probes present on the CATMA array, only those corresponding to nuclear genes annotated at TIGR were used in this study (21 788 uniques genes). Among these, only genes which had the same expression profile between the two biological replicates were considered.

**Figure 12:**
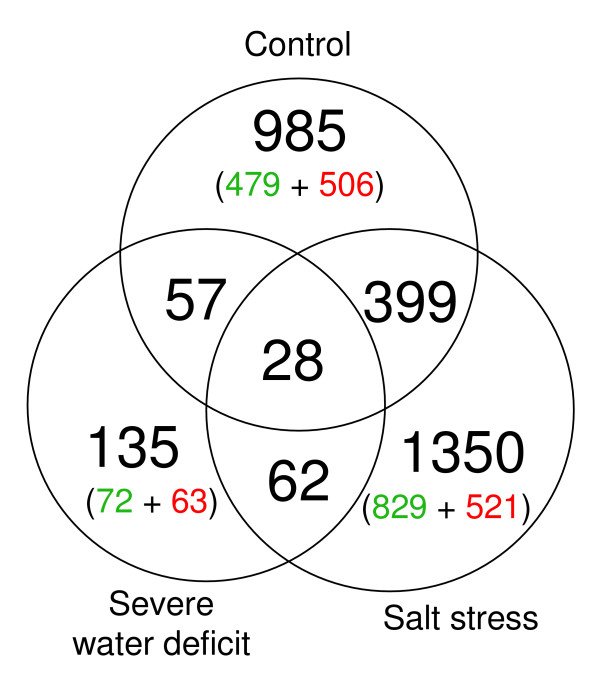
**Number of differentially expressed genes between *esk1-5 *and wild type**. The Venn diagram shows the overlap between differentially expressed genes under control condition, severe drought and salt stress (individually determined, table 2). The genes which were over-expressed in wild type are indicated in green and the genes over-expressed in the *esk1-5 *mutant are indicated in red.

The Venn diagram (Figure 12) shows the number of unique genes which were differentially expressed (induced or repressed more than 1.5 fold) between the Col-0 wild type and the *esk1-5 *line, for the 3 conditions tested: control, drought or salt stress. The overlaps represent the numbers of differentially expressed genes which were common between 2 or all 3 conditions.

**Figure 13:**
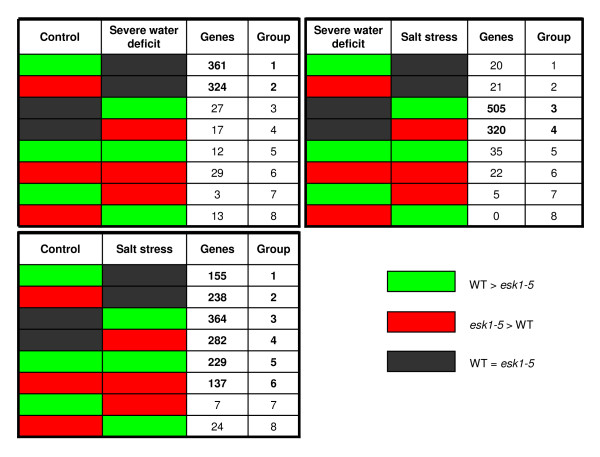
**Differential gene expression between *esk1-5 *and wild type**. The green cells represent genes that were over-expressed in the Col-0 line (and under-expressed in the *esk1-5 *mutant line). The red cells represent genes that were over-expressed in the *esk1-5 *mutant line (and under-expressed in the Col-0 line). The black cells represent genes that were not differentially expressed between the Col-0 and the *esk1-5 *mutant line. The third column in each table shows the number of genes in each group (fourth column). Top-left table: comparison between the control and the water deficit conditions. Bottom-left table: comparison between the control and the salt stress conditions. Top-right table: comparison between the water deficit and the salt stress conditions.

Under control conditions, the mutation led to changes in expression of 4.5% of the nuclear transcriptome. When drought or salt stress conditions were induced, expression of 0.6% and 6.2% of the nuclear transcriptome changed, respectively. Thus, a striking finding of our study is that the highest number of similarly expressed genes was found in the wild type and mutant under drought conditions: only 135 genes with an AGI (Arabidopsis Genome Initiative) code were differentially expressed between wild-type and *esk1-5*, compared to 985 under control conditions and 1350 following salt stress. In Figure 13, the profile of the differentially expressed genes is shown, comparing two treatments at a time. In the control vs. drought treatment, it is striking that the expression of the two largest groups of genes which were over-expressed in wild type or mutant in control conditions, then became equal under drought (groups 1 and 2). Whereas, only a small number of genes were equally expressed in the two genetic backgrounds in control conditions but differentially expressed under drought (groups 3 and 4). The situation was much more complex for the control compared to salt stress conditions: groups 1, 2, 3, 4 but also 5 and 6 all include a significant number of genes. Only two groups (7 and 8) are under represented, those where genes are over-expressed in wild type under control conditions and over-expressed in the mutant under salt stress and vice versa. Almost half of the genes were equally expressed under control conditions but changed expression following salt stress (groups 3 and 4), meaning that they were only affected by the mutation under salt stress but not in control conditions. Groups 5 and 6 include a large number of genes that were over-expressed either in wild type or in mutant under control and salt conditions, and thus may reflect a differential expression profile specific to the mutant rather than a general stress response. Finally, when drought and salt stress are compared, groups 3 and 4 are numerically the most important: most of the genes which showed differential expression between the two stresses are not affected by the mutation in drought conditions, but by salt stress conditions. Thus this subgroup of genes may have been specifically induced in the mutant by salt stress.

**Figure 14:**
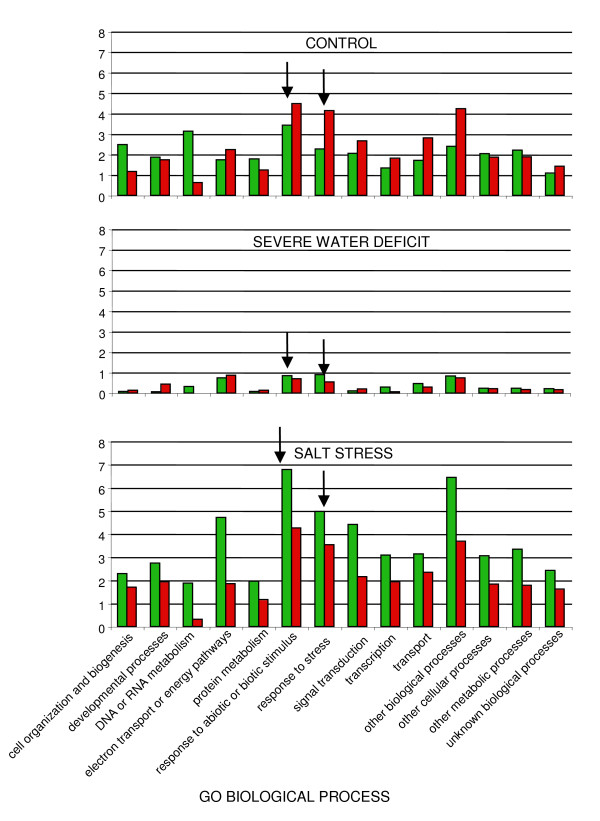
**Functional categories in the transcriptome of *esk1-5 *vs. wild type – Biological process**. Differentially expressed genes between *esk1-5 *and wild type, under control, severe water deficit and salt stress were classified into functional categories according to the GO "Biological process" at TAIR. Green bars show the percentage of over-expressed genes in wild type and red bars show the percentage of over-expressed genes in mutant, compared to the whole genome annotation. Arrows indicate the functional categories related to stress response.

**Figure 15:**
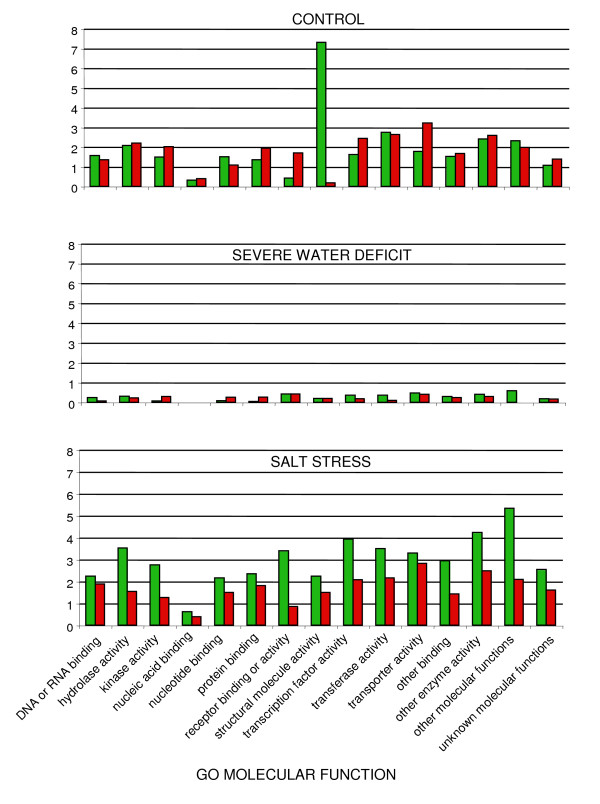
**Functional categories in the transcriptome of *esk1-5 *vs. wild type – Molecular function**. Differentially expressed genes between *esk1-5 *and wild type, under control, severe water deficit and salt stress were classified into functional categories according to the GO "Molecular function" at TAIR. Green bars show the percentage of over-expressed genes in wild type and red bars show the percentage of over-expressed genes in mutant, compared to the whole genome annotation.

In figures 14 and 15, genes which were differentially expressed between wild type and *esk1-5 *mutant under the three conditions tested, were categorised according to their function, based on the Gene Ontologies (subsets of the GO: Biological process and Molecular function). The results are presented as the percentage of the total number of genes in the whole genome found in each category. In this analysis, we did not include the last four categories of genes belonging to the non-specific classes: "other biological processes", "other cellular processes", "other metabolic processes" and "unknown biological processes". Once again, we observed a small difference in the number of differentially expressed genes between wild type and mutant under water deficit stress. The differences were higher following salt treatment compared to the control conditions. Most of the genes that were differentially expressed in wild type due to the salt treatment were over-expressed. With regards to the 'Biological process' involved (Figure 14), under control and salt stress conditions, we observed that two categories: "Response to abiotic or biotic stimulus" and "Response to stress" were over represented. Under control conditions, genes from these categories are over-expressed in the *esk1-5 *mutant and under salt stress conditions, they are over-expressed in wild type. Considering the 'Molecular function' '(Figure 15), in the wild type, under control conditions, we observe a pronounced over-expression of the group entitled "structural molecule activity", mostly due to ribosomal proteins (data not shown). Thus in summary, following salt treatment, the transcriptome is different between wild type and *esk1-5*, but none of the gene categories identified were of significant interest regarding stress response.

We set up a screen to identify a list of the genes that were either not expressed at all or weakly expressed (around the background) in wild type but over-expressed or highly repressed in the *esk1-5 *mutant, in the three conditions (Additional file 1). The experimental background was set at around 7.5 and an intensity of less than 9 corresponded to low expression. In the following section, we only refer to genes that can be discussed in an *eskimo1 *context.

Among the genes that are strongly **over-expressed in *esk1-5 *in control conditions **and weakly expressed in wild type, we selected: GSTF12, a member of glutathione S-transferase gene family among which each gene shows a particular inducibility by stress [33]; CAX3 (Calcium Exchanger-3) involved in ion homeostasis [34]; DFR (Dihydroflavonol Reductase) which is involved in the flavonoid biosynthetic pathway and also responds to environmental conditions [35]; ATHB-7 (Homeobox Leucine Zipper-7) a transcription factor induced by water deficit and by ABA [36]; PR2 a Pathogenesis-Related gene involved in the acquisition of systemic resistance [37]: all these genes are potentially involved in general defence responses. Other genes identified are noteworthy for their implication in development, such as MBP2 (Mirosinase-binding protein-2 [38]), or metabolism, such as MAM-3. (Methylthioalkylmalate-3 [39]). RDR-2, a RNA-dependant-RNA-polymerase-2, is involved in chromatin modifying via small-interfering RNA pathway [40]. NIA-1, the Nitrate reductase-1 and NCED-4, a nine-cis-epoxy-carotenoid-dioxygenase (or CCD4, Carotenoide Cleavage Dioxygenase) obtained lower scores (respectively r = 1.92 and 1.60) but are also worth mentioning.

Among the genes that are **under-expressed in the *esk1-5 *mutant in control conditions**, GLP-3, a germin-like protein obtained a very high score (r = 6.29). Scores were lower but still significant for potentially interesting metabolism genes: KCS-8, a 3-ketoacyl-CoA synthase; a FLS or Flavonol synthase and CSD-2, a superoxide dismutase. Two genes might be involved in signal transduction: FLA2, a fasciclin-like arabinogalactan which shows a rapid decrease in response to ABA [41] and PRP4 which is a structural Proline-rich protein.

It is striking that **under drought conditions only 11 genes were seen to be over-expressed or repressed **in the *esk1-5 *mutant and none of these are expressed more than 5 times. Nevertheless, NIA1 appears to be over-expressed. A gene encoding XTR3, which belongs to a Xyloglucan endotransglucosylase/hydrolase family [42], is repressed in the mutant but there is no evidence that this particular member plays a role in the cell wall construction and we did not observed any difference between the cell wall composition of wild type vs. *esk1-5 *and *esk1-4*, based on Fourier-Transform Infrared microspectroscopy profiles [43] (data not shown). APT3, SAD1 and/or KAT5 (one GST hybridises with SAD1 and KAT5) are also repressed in *esk1-5*. A mutation in SAD1 (Super Sensitive to ABA and Drought) led to hyper-reactivity to drought stress and ABA [44]. KAT5 encodes a putative 3-ketoacyl-CoA thiolase. APT3 encodes an Adenine phosphoribosyltransferase and may contribute to cytokinin metabolism [45].

The situation is more complex **under salt stress**: 61 genes were over-expressed and 107 genes are under-expressed **in the *esk1-5 *mutant**. NIA1 is strongly **over-expressed **in the three conditions. We also noticed some genes that are known to be induced by low temperature, dehydration and ABA: LTI30 (previously called XERO2) belongs to the dehydrin or LEA (Late Embryogenesis Abundant) family [46,47], RD29B or Responsive to Dehydration29B is also known to be induce by salt [48,49]; COR78 or Cold Regulated78 or RD29A [50]. DREB2A (Dehydration Responsive Element-Binding protein2A) which is not induced by ABA is also over-expressed in the mutant [51]. Among the genes **repressed in *esk1-5 *compared to wild type under salt stress**, some were also repressed under control conditions: GLP3 obtained a very high ratio (r = -7.06); AT2G10940 and AT2G15090 are annotated as being involved in the storage and metabolism of lipids, respectively; AT1G04800 is annotated as being involved in N-terminal protein myristoylation, a mechanism that could play a role in regulating signals produced by salt stress [52]. Several other interesting genes are repressed in the mutant: βCA1, a carbonic anhydrase-1 (in plants, Carbonic anhydrases are involved in the fixation of inorganic carbon); UBC6 contains an Ubiquitin conjugating (UBC) domain and Plasma membrane intrinsic protein1;5 (PIP1;5) and Tonoplast intrinsic protein2;2 (TIP2;2) are both aquaporins. Aquaporins are involved in water uptake from the soil and root hydraulic conductivity [53]. ABA2 encodes a xanthoxin dehydrogenase involved in the synthesis of ABA [54].

## Discussion

### Cold response

The *Eskimo1 *mutation was first identified as a mutation conferring frost survival without an acclimation period [30]. We did not observe this type of freeze tolerance in our experimental system, i.e. with plantlets grown in soil, either with the original *esk1-1 *mutant line, or three independent insertional mutant lines. Xin and Browse, however, carried out frost tests *in vitro *and we did them in soil, which might explain the reason for the phenotypic differences observed. We applied abiotic stress to plants in soil rather than *in vitro *because it is closer to field conditions. Our experimental system and results are more similar to those of Reyes-Diaz *et al*. [55], who worked with plants in pots, at the 10–15 leaf development stage and did not observe any difference in freezing tolerance without acclimation between the *esk1-1 *mutant line and its wild type genetic back-ground. They reported that both the wild type and the *esk1-1 *mutant can tolerate freezing only after a cold acclimation period and that without acclimation, the two genotypes avoid freezing by delaying or preventing frost damage. Here, we also clearly showed that *ESKIMO1 *mutants are more tolerant to freezing but only after acclimation (Table 4).

### Drought and salt responses

In a recent article, Xin and collaborators found that the *esk1-1 *mutation was not involved in drought and salt stress responses [32]. Originally, we selected *esk1-6 *as a candidate gene after an *in silico *analysis because it has sequence similarities with a maize EST that changes expression in response to cold treatment. We screened for drought and cold response independently and selected the *ESKIMO1 *mutant in both screens. We observed significant differences in the response to mild drought, water starvation and cold stress between soil grown wild type and *esk1-6 *at the 6^th ^leaf stage (Figures 2, 3; table 1, 2, 3). No differences in root growth were observed *in vitro *following salt and osmotic stress (Figure 4). The two independent mutant lines *esk1-4 *and *esk1-5 *showed similar phenotypes (Figure 2, 3, 4, 5, 6, 7, 8, 9, Table 1, except for the PRL *in vitro*, Figure 4) and responded to stress the same way. Therefore, the phenotype differences can be confidently assigned to the *ESKIMO1 *mutation. The phenotype of the *esk1-6 *mutant which has an insertion in the promoter region is slightly different (Figure 2, Figure 3). Progeny tests showed that the *esk1-6 *mutation is recessive (Table 2). Thus the slight differences observed between *esk1-6 *and *esk1-4 *and *esk1-5 *are most likely due to the different genetic background and/or changes in *ESKIMO1 *expression. In summary, the general characteristics observed for the three mutant lines were highly similar and can be clearly attributed to the mutation in *ESKIMO1*.

We showed that in standard and drought conditions, the mutants' transpiration rate was lower than that of the wild type. We suspect that stomatal conductance is lower in the mutant which is supported by the result showing slower "Cut Rosette Water Loss". However, we also determined that the transpiration results cannot be explained by reduced stomatal density, which was actually higher in the mutant.

### Water Use Efficiency

Since the *esk1 *mutants are smaller than wild type plants, their water needs are expected to be lower, but the parameter which is of biological relevance is water required per biomass unit. WUE was assessed by measuring CO_2 _consumption and H_2_O release with a portable gas exchange system. Our results clearly show that the WUE of the *esk1-5 *mutant is higher than the wild type. Due to the small size of the mutant leaves, it was not possible to assess to the gas exchange under stress conditions with the previous system. In addition, this type of measurement is taken at selective time point so that the results can vary depending on the metabolic state of the leaf at the measurement time. Thus we choose to use an alternative method based on carbon isotope discrimination (δ^13^C). Carbon fixation during photosynthesis discriminates against the heavy carbon isotope (^13^C) [56]. Because WUE is highly correlated to carbon isotope discrimination, δ^13^C can be measured as a reliable indicator of WUE. This correlation has been observed in wheat [57] and in *Arabidopsis thaliana *[58]. The results showed that the two allelic mutants have a higher WUE (Table 5) than the wild type. We also observed that the WUE of both wild type and mutant plants improved slightly following drought treatment but that salt treatment does not seem to affect WUE. Because δ^13^C reflects the isotope discrimination signature for the life-time of the plant, it is not surprising that a three day stress did not affect this measure. It is more surprising, however, that we observed a general tendency for an improvement in the WUE, in the three genotypes, under drought conditions, after only 4 days of reduced soil water content. All together, these results show that the *esk1 *mutant has an improved WUE and a higher photosynthetic rate. In a review article, Parry *et al*. [59] postulated that this is achieved in three possible ways: a CO_2 _concentrating mechanism, increased mesophyll conductance or increased performance of rubisco (D-ribulose 1,5-bisphosphate carboxylase/oxygenase).

### Transcriptome analysis

Transcriptomic analysis showed that under control conditions 985 genes are differentially expressed between wild type and the *esk1-5 *mutant (Figure 12) but only 57 of these genes are still differentially expressed in drought conditions. It can be clearly seen in figures 12, 13, 14 and 15 that the transcriptomes of the wild type and mutant are similar under mild water deficit stress, but not in control conditions. We hypothesise that the mutation in the *ESKIMO1 *gene leads to a physiological response preparing the plant for drought stress, explaining why some genes involved in stress responses were already expressed during the watering regime. In line with this theory, a large proportion of genes which are differentially expressed between the wild type and mutant were assigned to functional categories related to defence and environmental interactions. We propose that the other functional categories differentiating the wild type from the mutant are a consequence of a perturbed metabolism in the mutant. The proportion of differentially expressed genes is larger under salt stress than under control condition. Even if there is a lot of crosstalk between abiotic stresses like drought, cold, osmotic and salt stress, the *ESKIMO1 *gene appears to specifically mimic water depletion. Both drought and salt stress sensed by the plant will progressively lead, depending on their intensity, to osmotic stress caused by cellular dehydration [6]. Drought also has a mechanical stress component due to soil hardening [9], and salt stress has an ionic component which may be toxic and induce specific genes. The fact that the "structural molecule activity" category (ribosomal proteins) is repressed in the mutant may also mimic abiotic stress: down-regulation of genes involved in protein synthesis was described in *Populus euphratica *in response to salt stress [60] and in maize in response to osmotic stress [61]. Also of note, the "protein biosynthesis" category is down-regulated and "carbon utilisation" is up-regulated in citrus in response to gibberellins [62]. Genes related to abiotic stress, mainly water response, were differentially expressed in this study.

Two genes annotated as transcription factors were reported to be highly over-expressed in the *esk1-1 *and *esk1-4 *mutants in Xin *et al*.'s article and were also identified in the *esk1-5 *mutant in our control conditions. Plants in Xin's experiment were grown *in vitro *and harvested at 14 days. In our conditions plants were grown on propagation plugs and harvested a week after bolting. As a consequence, we can postulate that these two genes, AT1G18710 and AT2G46680, are major contributors to the expression of the phenotype in the *eskimo1 *background.

We found that one of the two nitrate reductase genes, NIA1, is highly over-expressed in the mutant in the three conditions. The other NR gene, NIA2 is expressed in both *esk1-5 *and the wild type, but its expression is slightly higher in the mutant under control and drought stress conditions. This may reflect improved carbon assimilation in the *esk1 *context, regardless of the environmental conditions, because this process has been correlated with NR activity [63]. Elsewhere, NR was found to be required for stomatal closure in an ABA-dependant pathway, by generating the signalling molecule nitric oxide [64]. This mechanism could maintain the stomata closed in the *esk1-5 *mutants depending on nitric oxide signalling.

We also observed that a number of genes that play or that may play a role in general defence responses are over-expressed in *esk1-5 *in control conditions. These genes are listed in the supplementary material and are described in results section. Nevertheless, none of the known key players in stress response such as the transcription factors DREB2A, DREB2B and CBF4, responsive genes RD29A and RD29B, or genes involved in salt response from the Salt Overly Sensitive family... were found to be differentially expressed. Thus the low evapo-transpiration stress symptom of the *esk1-5 *mutant under control conditions may reflect a different mechanism than that typically induced by the bulk of stress responsive genes. We observed that three aquaporins are repressed in the mutant: a Tonoplast Integral Protein (AT3G16240 or DELTA-TIP), and two Plasma membrane Intrinsic Proteins (AT4G23400 or PIP1;5, AT3G54820 or PIP2;5). One PIP is over-expressed in the mutant in control conditions (AT3G61430 or PIP1A). Thus, an overall hydraulic disruption in the mutant genotype might be a signal for stomatal closure. One aquaporin (AQN1) in *Nicotiana tabacum *is located in the chloroplast membranes and facilitates CO_2 _diffusion and assimilation [65]. However, more experiments are needed to pinpoint the precise role of the aquaporins differently expressed between wild type and *esk1-5 *mutant. Several genes that are usually associated with drought stress are differentially expressed between wild type and the *esk1-5 *only under salt stress: RD29A and RD29B, DREB2A and LTI30. These four genes also gave much higher hybridisation signals on the CATMA microarray under drought stress than under control conditions (the background noise was around 7.5 and the four genes showed signals between 10.07 and 13.40 under drought stress). This suggests that their expression is affected by drought stress but they are highly induced by salt only in the *esk1-5 *mutant background.

### General discussion

Our results can be discussed in light of those of Xin and coworkers, who carried out water starvation tests on wild type and mutant plants (*esk1-1*) growing in the same pots, and concluded that the mutation was not associated with an increased ability to survive drought or salt stress. We observed that wild type consumes more water than the mutant lines (*esk1-4 *and *esk1-5*), so it is not surprising that in the same pot, the wild type would first use up the available water, exhausting the substrate for all the plants. Once a critical soil water potential is reached, *eskimo1 *mutants are not different from wild type. We propose that the *eskimo1 *mutants take more time to exhaust the water from a given substrate and convert it to biomass more efficiently. Another significant difference between our findings and those of Xin and collaborators is that they did not observe a difference in the effect of salt on wild type and mutant plants (*esk1-1*). Again, the experimental conditions were very different in the two studies. Their salt response experiment was carried out *in vitro *with seedlings three days after germination, and indeed, we also failed to observe any difference in the response to salt by young plants *in vitro*. We hypothesise that the results we obtained with mature plants in response to salt, i. e. the wild-type but not the mutant leaves presented lesions close to the meristem, is a consequence of differences in the plant water economy. It is likely that, in wild type the water-salt solution was pumped from the soil faster than in the mutant and caused damage to the plants.

It also seems that the phenotype we observed in our large mutant screen was not, in the strict sense, a response to drought: the *eskimo1 *mutant uses less water which means that the substrate will dry more slowly. Therefore, rather than being tolerant to drought *per se*, the mutant can overcome a water deficit period more easily than wild type. Nevertheless, our phenotype screen is accurate because we selected the *eskimo1 *mutant due to its severely disturbed response to drought stress and it would not have been selected by observing *in vitro *responses to osmotic or salt stress (our results).

## Conclusion

Based on our findings, we conclude that the *ESKIMO1 *gene plays a major role in whole plant water economy. We determined that the *eskimo1 *mutation leads to a loss in fitness, but in drought conditions most of the wild type died whereas the mutant lines keep producing seeds. We are currently generating transgenic lines in which the *ESKIMO1 *gene will be inactivated in response to abiotic stress in order to minimise this fitness cost of the mutation but maximise survival and WUE under drought stress. We are also searching for natural alleles of *ESKIMO1 *that could change the expression of the gene and/or the functionality of the protein. Condon *et al*. reported the release of new varieties from breeding selection for δ^13^C to improve WUE and grain yield in wheat [66]. *ESKIMO1 *has homologous genes in numerous species. It is tempting to speculate that allele selection or manipulation of *ESKIMO1 *in crops could improve WUE.

Plant response to abiotic stress is a complex trait divided among distinct but cross talking pathways. Expression of the regulators of the genes involved in the response is itself tightly regulated [5,67]. We are particularly interested to know if the *ESKIMO1 *gene is a negative regulator of stress response as postulated by Xin *et al*. [32] or if the induction of abiotic stress genes in the mutant line(s) is a secondary consequence of the plant water status due to a water uptake deficiency.

## Methods

### Plant lines

Mutant lines in the AT3G55990 gene were obtained either from the INRA Resource Centre for *Arabidopsis thaliana *Genomics : *esk1-6 *in the WS genetic background [68], or from The Salk Institute in the Col-0 genetic background: SALK_078275 (*esk1-4*) and SALK_089531 (*esk1-5*) [69]. The WS and Col-0 lines used were from the INRA Versailles Resources Centre: 530AV and 186AV.

### Drought, cold and salt treatments

#### • Monitored stress applied in propagation plugs

Arabidopsis plants were grown following standard procedures established by Loudet *et al*. [70]. Seeds were stratified for 4 days in a 0.1% (w/v) agar solution at 4°C in the dark. Germination occurred 2 days after sowing on propagation plugs (4 cm height × 4 cm radius – 70% blond peat, 20% perlite and 10% vermiculite, Fertiss^®^). Plants were grown under long day conditions with a 16 h photoperiod, in a controlled environment chamber (22°C, 70% RH, PPFD approximately 150 μmol m^2 ^s^-1^) and watered with nutritive solution as described in Bouchabke *et al*. [71]. The relationship between soil volumetric water content and soil suction was previously assessed [71].

During plant growth prior to starting the stress experiments the propagation plugs were saturated with nutritive solution (100% at t0). During the stress experiments, however, propagation plugs were weighed daily from t0. Once the target saturation was reached, this was then maintained for the duration of the experiment. For controls, soil water content was fixed at 60% of substrate maximal water content (SMWC). The mild water-deficit treatment was fixed at 30% SMWC whereas severe water-deficit corresponded to 20% SMWC. Two approaches were used to control the saturation level, either an averaged or an individually monitored stress, as indicated in Table 2. For the averaged determination method, the weight of ~10% of the propagation plugs was measured and all the plugs adjusted with the average volume calculated to reach the targeted saturation. For the individual determination method each propagation plug, within a set watering regime, was maintained at the same saturation level based on its actual weight. In this way, the substrate saturation of all the genotypes within a watering regime was identical regardless of their water consumption. The individual determination, which is more labour intensive was employed for the experiments where result reproducibility was most likely to be affected by slight differences in the stress imposed, namely the transcriptome and carbon isotope discrimination analyses (Table 2, Figure 11). To measure integrative parameters such as TLA or CRWL, stress was monitored using the averaged determination of the propagation plugs. Experimental start points varied depending on the phenotype examined; for TLA and CRWL t0 was when leaf number 6 emerged (growth stage 1.6 according to Boyes *et al*. [72]), For transcriptome and carbon isotope discrimination, treatments were applied from floral bud emergence onwards (growth stage 5.10 according to Boyes *et al*. [72]).

Salt stress experiments were conducted in parallel to those for drought stress. The propagation plugs for treated plants were first reduced to 60% saturation before the salt stress was applied by watering with 0.5× nutritive solution supplemented with 150 mM NaCl. In this way, plants were subjected to each stress for the same time period.

#### • Water starvation

Plants were cultured in peat moss in pots (length 60 mm, width 65 mm, height 60 mm), filled equally with a homogeneous non-enriched compost (Terf^® ^Substrat: 37% blond peat, 60% brown peat, 10% volcanic sand). The pH of this compost was stabilised between 5.5 and 6.1.

Plants were grown in the same environmental conditions as described above. Progressive drought was applied on 9 randomly selected one-month-old plants of each line (stage 5.10 according to Boyes *et al*. [72]) by stopping watering. As a control, the same number of plants of each line was grown under standard irrigation conditions and watered twice a week. Pictures of the canopy were taken at day 0, 6 and 10 of stress exposure to calculated the Photosynthetic Leaf Area. PLA is equal to TLA minus chlorotic areas.

#### • Cold treatments

Seeds were stratified as described earlier (Monitored stress applied in propagation plugs). Then a large-scale screen to test cold tolerance was performed as follows: rows of plants were sown in square pots containing organic substrate and irrigated with mineral nutrient solution once a week and watered every four days. Plants were grown in the greenhouse for 14 days at which time they had reached the 6–8 leaf stage (stage 1.04 according to Boyes *et al*. [72]). Plants were then transferred to a growth chamber at 5°C under 12 h photoperiod, 70 μM m^-1^s^-1 ^light intensity and 70% relative humidity for 7 days. Acclimated plants were then exposed to freezing temperatures of -8°C for 48 h. After this cold treatment, plants were put back in the greenhouse. Tolerance to freezing was determined by evaluating the percentage of viability after freezing exposure: viable and dead plants were counted and the percentage viability was calculated. Four rows of the mutant and two rows of the reference strain were put in each square pot to optimise viability comparisons by reducing undesirable environmental variation. At least 400 plants were tested per line. Parallel experiments were carried out without the acclimation period.

#### • Salt treatments

Plants were grown in pots as described for the water starvation experiment. For 12 days, 3 × 10 plants of each line were watered every two days with a concentrated saline solution (NaCl 200 mM). Every three watering cycles plants were watered with non-saline water. Results were compared with the same number of plants grown with standard irrigation. Pictures were taken at day 0, 6 and 10 of stress exposure.

For culture on agar plates, seeds were sterilised, stratified four days at 4°C in the dark and then transferred onto 3 × 10 plates of solid medium [73]. Plants were cultivated in growth chambers under long-day conditions (16 h/d) at a photon flux density of 120 μmol m^-2 ^s^-1^. Temperature (21°C) and relative humidity (70%) were constant in the growth chamber. For *in vitro *salt stress induction, the solid medium was supplemented with 3 different NaCl concentrations (100; 150; 200 mM). The same number of standard media plates was prepared as a control. For leaf development studies, 9 × 3 seeds from each line were randomized at regular intervals inside the plate. For root development analysis, six seeds were placed (from each genotype) on each plate, close to one of the edges. Plates were laid horizontally for 48 hours and then placed vertically in a rack, with the seeds at the top. All plates were collected at day 12 and scanned with a desktop scanner (Epson scan Photo 4990) using the "transparent object" mode at 300 dpi.

#### • Osmotic treatments

Osmotic stress was induced following the same protocol as for salt stress in solid media, but supplemented with 60 or 75 mM mannitol.

### Fresh weight, dry weight, water loss and transpiration

Fresh weight (FW) was obtained by harvesting and weighing freshly cut rosettes (stage 3.70 to 3.90 according to Boyes *et al*. [72]).

Rosette dry weight was recorded after 48 h at 75°C in a dry oven.

Relative Water Content (RWC) was calculated according to the formula: [(FW-DW)/DW] × 100.

Cut Rosette Water Loss (CRWL) indicating the amount of water lost from freshly cut tissues during the first 60 minutes, was determined by harvesting and weighing freshly cut rosettes. Rosettes were maintained in the growth chamber conditions then weighed every 10 minutes. CRWL was then calculated as the ratio between water loss and plant initial fresh weight, expressed in %.

To assess transpiration *in planta *the rosettes of 6 plants per genotype and per soil water treatment were isolated from the soil with a plastic film. The entire propagation plug was also covered with plastic film preventing any soil evaporation. Propagation plugs without rosettes were also included in the experiment to assess water evaporation from empty propagation plugs. Two hours after the beginning of the light period, plants were weighed every two hours for 36 hours. Transpiration per unit of dry weight was then calculated as the ratio between transpiration (weight of the propagation plug at tx time, minus weight of the propagation plug at t0, minus evaporation from empty propagation plugs) and the plant dry weight.

### Stomatal density

The number of stomata per leaf area was determined on the 10th or 11th leaf of five plants grown in control conditions in short days, in the greenhouse (stage 1.13 to 1.14 according to Boyes *et al*. [72]). Leaves were fixed in ethanol/acetic acid (3/1) for one hour, and then washed three times with pure water. After this step, they were bleached in NaOH 8 M for one hour, then washed three times with pure water. The surplus water was wiped away. The leaves were mounted in a 0.1% calcofluor solution and observed with a F.I.S.H (Fluorescent In Situ Hybridization) microscope at a 350 nm wavelengh (UV light), magnification 12.5×. Six pictures of each leaf were taken on the whole leaf surface excluding the central nervure. The stomata were counted with ImageJ software using the "cell counter" plugin.

### Gas exchange measurements

Gas exchange was measured from one leaf of three independent plants of each genotype (Col-0 and *esk1-5*) using a portable gas exchange system (Li-6400; LI-COR) with a standard leaf chamber (6400-40 with red and blue LED light source; LI-COR). Leaf chamber conditions were 400 μmol mol^-1^CO_2_, 21% O_2_, 51.47% relative humidity, 22°C and photosynthetic photon flux density (PPFD) of 500 μmol m^-2 ^s^-1 ^with 10% of blue light. Leaves were kept under this condition for approximately 30 min until parameters were stabilized before recording. Because Col-0 and especially the *esk1-5 *leaf were too small to fill the entire area of the leaf chamber, the portion enclosed parts of leaves were marked. The leaves were then cut from plants, scanned and the total leaf area (which had been enclosed in the chamber) was evaluated by image analysis using a similar procedure as described in the data processing section below. Net photosynthesis [74] and transpiration (ET) were calculated by using equations derived by Caemmerer and Farquhar [75]. WUE was estimated by the net photosynthesis/evapo-transpiration ratio (NP/ET).

### ∂^13^C assessment

After 7 days of individual determination of treatments, frozen samples were lyophilized and then ground. For each sample, about 1 mg of powder was transferred into tin cups (Courtage analyse service, Mont Saint-Aignan, France) and analysed in an elemental analyser (NA-1500, Carlo Erba, Milan, Italy) coupled to an Isotope Ratio Mass Spectrometer (VG Optima, Fison, Villeurbanne, France). Carbon isotope compositions were calculated as deviations of the carbon isotope ratio (^13^C/^12^C, called *R*) from international standards (Pee Dee Belemnite) according to Farquhar *et al*.:[56] δ^13^C = |10^3 ^[(*R*_sample _- *R*_standard_)/*R*_standard_]|.

### Transcriptome

For the transcriptome analysis, RNA was extracted with the RNeasy extraction kit from Qiagen^® ^including the DNase treatment. Microarray analysis was carried out at the Unité de Recherche en Génomique Végétale (Evry, France), using the CATMA array [76,77], containing 24,576 Gene-Specific Tags from Arabidopsis. RNA samples from two independent biological replicates were used. For each biological replicate, RNA samples for a condition were obtained by pooling RNA from 3 plants. For each comparison, one technical replicate with fluorochrome reversal was performed for each biological replicate (i.e. four hybridisations per comparison). RT on RNA in the presence of Cy3-dUTP or Cy5-dUTP (Perkin-Elmer-NEN Life Science Products), hybridisation of labelled samples to the slides, and the scanning of the slides were performed as described in Lurin *et al*. [78]. Microarray data from this article were deposited at Gene Expression Omnibus (; accession No. GSE10384) and at CATdb (; Project RA06-02_StayGreen) according to the "Minimum Information About a Microarray Experiment" standards.

### Data processing

Rosette surface measurements were performed as followed: rosette surfaces were selected using Photoshop^® ^software (selection/colour range) and saved as .tif files. Files were then opened with ImageJ software and transformed to 8-bit. The global scale was set with the help of a ruler in the initial picture, the threshold was adjusted to optimise the selection and the area was measured. Statistical analyses were performed with Statgraphics^® ^software. We used the term Total Leaf Area (TLA) for a green rosette and Photosynthetic Leaf Area for a damaged rosette. PLA corresponds to the area of non-damaged leaves.

For the transcriptome analysis, experiments were designed with the statistics group of the Unité de Recherche en Génomique Végétale. Statistical analysis was based on two dye swaps (i.e. four arrays, each containing 24,576 GSTs and 384 controls) [78]. Controls were used for assessing the quality of the hybridisation, but were not included in the statistical tests or the graphic representation of the results. For each array, the raw data comprised the logarithm of median feature pixel intensity at wavelengths 635 (red) and 532 nm (green). No background was subtracted. In the following description, log ratio refers to the differential expression between two conditions. It is either log2 (red/green) or log2 (green/red) depending on the experimental design. Array-by-array normalisation was performed to remove systematic biases. First, we excluded spots that were considered badly formed features. Then, we performed global intensity-dependent normalisation using the LOESS (locally weighted scatterplot smoothing) procedure to correct the dye bias. Finally, for each block, the log ratio median calculated over the values for the entire block was subtracted from each individual log ratio value to correct print tip effects on each metablock. To identify differentially expressed GSTs, we performed a paired *t*-test on the log ratios, assuming that the variance of the log ratios was the same for all genes. Spots displaying extreme variance (too small or too large) were excluded. The raw p-values were adjusted by the Bonferroni method, which controls the FWER (Family-Wise Error Rate). We considered genes as being differentially expressed with a FWER of 5%. We used the Bonferroni method (with a type I error equal to 5%) in order to keep strong control of false positives in a multiple-comparison context [79]. A manual clustering step was carried out only considering GSTs with the same expression pattern in the two biological replicates. In this manuscript, any differentially expressed GST that hybridises with two genes (genes with an identification number in The Arabidopsis Information Resource or TAIR) is accounted as two distinct genes. A Perl script was developed to select genes which were highly expressed or highly repressed between wild type and mutant, and expressed at around the background level in wild type or mutant (with a high log2 (ratio) range and with a log2 (red or green intensity values) of around 7.5). Results are presented in the Additional file 1. Analysis of the functional categories of genes according to the Gene Ontology and the whole *Arabidopsis thaliana *genome annotation was made with the TAIR GO annotation tool  September 3^rd^, 2008.

## Authors' contributions

OBC carried out the mutant screening, physiological drought tests and wrote the manuscript. MQ, JSR and JT performed physiological drought and salt tests and participated in preparing the manuscript draft. MF participated in all the tests, except the cold response tests, and contributed to the manuscript draft and revisions. CG did the cold tests. AY did the transcriptome processing. DL performed physiological drought, osmotic and salt tests. FG did the transcriptome analysis. ET planned, analysed and wrote the cold test section of the manuscript. MDT contributed to the experimental design and all drought and salt tests as well as writing the manuscript and coordinating the project. All authors read and approved the final manuscript.

## Supplementary Material

Additional file 1 **Transcriptomic data mining.** Genes highly expressed, or highly repressed, between wild type and mutant, and expressed at around the background level in wild type or mutant (see Methods, Data processing), in control, drought or salt stress conditions. Click here for file
